# Effectiveness of Exogenous Fe^2+^ on Nutrient Removal in Gravel-Based Constructed Wetlands

**DOI:** 10.3390/ijerph19031475

**Published:** 2022-01-28

**Authors:** Liping Tian, Baixing Yan, Yang Ou, Huiping Liu, Lei Cheng, Peng Jiao

**Affiliations:** 1 Key Laboratory of Wetland Ecology and Environment, Northeast Institute of Geography and Agroecology, Chinese Academy of Sciences, Changchun 130102, China; tianliping@iga.ac.cn; 2 University of Chinese Academy of Sciences, Beijing 100049, China; 3 Jilin Provincial Engineering Center of CWs Design in Cold Region & Beautiful Country Construction, Changchun 130102, China; 4 College of Plant Protection, Jilin Agricultural University, Changchun 130118, China; lhp9827@163.com (H.L.); clxyjlau@163.com (L.C.); 5 College of Resources and Environment, Jilin Agricultural University, Changchun 130118, China; jiaopeng@iga.ac.cn

**Keywords:** Sanjiang Plain, ferrous iron, nitrogen removal, phosphorus removal, constructed wetlands

## Abstract

A group of microcosm-scale unplanted constructed wetlands (CWs) were established to evaluate the effectiveness of exogenous Fe^2+^ addition on ammonium nitrogen (NH_4_^+^-N), nitrate nitrogen (NO_3_^−^-N), and total phosphorus (TP) removal. The addition of Fe^2+^ concentrations were 5 mg/L (CW-Fe5), 10 mg/L (CW-Fe10), 20 mg/L (CW-Fe20), 30 mg/L (CW-Fe30), and 0 mg/L (CW-CK). The microbial community in CWs was also analyzed to reveal the enhancement mechanism of pollutant removal. The results showed that the addition of Fe^2+^ could significantly (*p* < 0.05) reduce the NO_3_^−^-N concentration in the CWs. When 10 mg/L Fe^2+^ was added and the hydraulic retention time (HRT) was 8 h, the highest removal rate of NO_3_^−^-N was 88.66%. For NH_4_^+^-N, when the HRT was 8–24 h, the removal rate of CW-Fe5 was the highest (35.23% at 8 h and 59.24% at 24 h). When the HRT was 48–72 h, the removal rate of NH_4_^+^-N in CWs with 10 mg/L Fe^2+^ addition was the highest (85.19% at 48 h and 88.66% and 72 h). The removal rate of TP in all CWs was higher than 57.06%, compared with CW-CK, it increased 0.63–31.62% in CWs with Fe^2+^ addition; the final effluent TP concentration in CW-Fe5 (0.13 mg/L) and CW-Fe10 (0.16 mg/L) met the class III water standards in Surface Water Environmental Quality Standards of China (GB3838-2002). Microbical diversity indexes, including Shannon and Chao1, were significantly lower (*p* < 0.05) in Fe^2+^ amended treatment than that in CW-CK treatment. Furthermore, phylum *Firmicutes*, family *Carnobacteriaceae,* and genus *Trichococcus* in Fe^2+^ amended treatments was significantly (*p* < 0.05) higher than that in CW-CK treatment. Fe^3+^ reducing bacteria, such as *Trichococcus* genus, belonging to the *Carnobacteriaceae* in family-level, and *Lactobacillales* order affiliated to *Firmicutes* in the phylum-level, can reduce the oxidized Fe^3+^ to Fe^2+^ and continue to provide electrons for nitrate. It is recommended to consider adding an appropriate amount of iron into the water to strengthen its purifying capacity effect for constructed artificial wetlands in the future.

## 1. Introduction

Diffuse pollution caused by agricultural production is one of the main causes of water pollution. In the United States, non-point source pollution accounts for more than 80% of total pollution; in Europe, more than 50% of river pollution is caused by agricultural non-point source pollution [1,2]. In China, non-point pollution from agriculture has also become the dominant source and key problem of water pollution control, as point source pollution has been effectively controlled [3]. Agricultural non-point source pollution sources are scattered and hidden, spatio-temporal patterns of the time and space of pollution are random and uncertain, and they are difficult to monitor and control [4,5]. Nitrogen and phosphorus enter surface water bodies through soil erosion, runoff, and leaching, thus leading to eutrophication and other environmental problems [6,7].

Nitrogen and phosphorus are the main pollutants of agricultural diffuse pollution. One of the important agricultural diffuse pollution mitigation measures is process control, which mainly includes ecological ditch, constructed wetland, and ecological concrete. Among the various methods, constructed wetlands (CWs) have been proven to be a convenient and cost-effective method for nutrient contaminated wastewater treatment [8,9]. In constructed wetlands, carbon is usually used as electron donors and nutrients for the denitrification process, and once it is insufficient, the microbial denitrification of the system will be negatively affected. Ferrous ion, as a reductive substance, could provide electrons for the reduction of nitrate, while phosphorus removal efficiencies are often high initially and then significantly decrease after a long term application for adsorption saturation of substrates in CWs [9].

Microorganisms could use Fe^2+^ as electron donors to convert NO_3_^−^ to N_2_. When electron donors such as organic carbon in the system are limited, Fe^2+^ could be used as electron donors to participate in the denitrification process to reduce NO_3_^−^-N [10]. In this condition, the essence of denitrification is the mutual conversion process of Fe^2+^ and Fe^3+^ [11]. Iron participates in denitrification in two ways, one is directly used in chemical reactions, the other is to promote denitrification microbial activity, which can reduce NO_3_^−^-N while oxidizing Fe^2+^. In addition, the reduction of Fe^3+^ may also participate in the anammox process and promote the emission of nitrogen oxides.

The coupling process of ammoxidation and dissimilation reduction (Feammox) of iron oxides under anaerobic conditions could be obtained in natural environments such as riparian wetlands and paddy soil [12]. Although many different forms of iron have been used for enhancing nitrogen removal in CWs, only a few focus on enhancing phosphorus removal. Through promoting non-biological processes, Fe^2+^ and Fe^3+^ will precipitate with PO_4_^3−^ due to solubility limitation, directly removing PO_4_^3−^ in wastewater. On the other hand, Fe^2+^ and Fe^3+^ will undergo hydrolysis reactions and various polymerization reactions through dissolution and water absorption to generate multinucleated hydroxyl complexes, thus indirectly lowering the PO_4_^3−^ of wastewater through adsorption and complexation.

The Sanjiang Plain, as an important commercial grain base in China, is faced with serious non-point source pollution. At the same time, rice is the main crop in the Sanjiang Plain, and the irrigation water is mainly from groundwater. Pan et al. [13] pointed out that Fe^2+^ level in the groundwater of the Sanjiang Plain was generally higher than that of Fe^3+^; the soluble iron concentration varied from 0.03 to 21.00 mg/L, with an average of 5.48 mg/L. Therefore, the abundant Fe^2+^ in the groundwater could be utilized to remove the nitrogen and phosphorus of farmland drainage in the Sanjiang Plain.

In constructed wetlands, most research is concentrated on the iron matrix (natural pyrite or sponge iron) to remove nitrogen [8,14,15]. Few studies have been conducted on the addition of Fe^2+^ in constructed wetlands for the simultaneous removal of nitrogen and phosphorus. Zhang et al. [16] investigated the effects of external Fe^2+^concentration (0, 5, 10, 50, 100, and 150 mg/L) on the removal of COD and different nitrogen and phosphorus forms in CWs. However, this research was aimed at high-concentration TN (185 mg/L), TP (20 mg/L), and COD (430 mg/L) industrial wastewater. There are a large number of depressions and degraded wetlands in the Sanjiang Plain, and if agricultural drainage with abundant Fe^2+^ could be used to transform them into constructed wetlands, it is expected to effectively solve the problem of regional surface source pollution control. Therefore, this study aims to (1) evaluate the impact of low level Fe^2+^ addition on the simultaneous removal rate of nitrogen and phosphorus in constructed wetlands, to (2) identify the bacterial community structure in biofilms from substrates under different Fe^2+^ concentrations, and to (3) determine the optimal Fe^2+^ input that can enhance the efficiency of constructed wetlands. 

## 2. Material and Methods

### 2.1. Experimental Description

**Figure 1:**
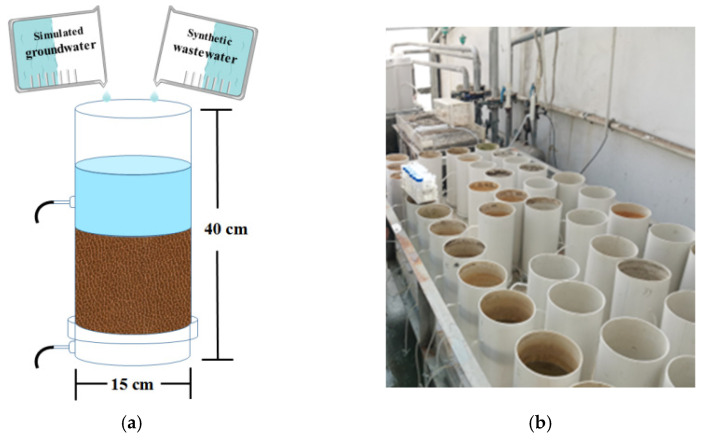
Schematic diagram (**a**) and photo of the CW microcosms (**b**).

A group of microcosm-scale unplanted constructed wetlands (CWs) made of polyethylene plastic with a thickness of 0.2 cm, a height of 40 cm, and 15 cm inner diameters were established. The specific structure and design are shown in Figure 1. The two outlets of the reactor are separated by 15 cm. The reactors are 15 cm apart with two water outlets from the bottom to the top. Each reactor is filled with 2.5 L of fine gravel (1–2 cm in diameter, the specific surface area is 0.03 g/m^2^, and the pore size is 82.99 nm). The reactors were placed in Greenhouse No. 3, Northeast Institute of Geography and Agroecology, Chinese Academy of Sciences, at a temperature of 25–30 °C.

### 2.2. Synthetic Wastewater and System Operation

The synthetic wastewater with a pH of 6.8–7.2 was from tap water. According to the nutrient concentration of paddy field drainage in the Sanjiang Plain, the concentrations of NH_4_^+^-N, NO_3_-N, and TP were set to 5 mg/L, 7 mg/L, and 1 mg/L, respectively. NO_3_^−^-N, NH_4_^+^-N, and TP in the synthetic wastewater were prepared with KNO_3_, NH_4_Cl, and KH_2_PO_4_, however, the existence of Fe^2+^ requires acidic conditions, and the simulated wastewater needed to be configured with tap water. Tap water contains a large number of chloride ions, which will quickly oxidize Fe^2+^ to Fe^3+^. A metal chelating agent can use the strong binding effect of complex agent molecules and metal ions. The metal ions are encapsulated inside the chelating agent to become stable compounds with higher relative molecular weight, thus preventing the precipitation of metal ions and controlling the amount of free ferrous ions in the solution. Therefore, the oxidation of Fe^2+^ can be controlled, to a certain extent, through the use of an appropriate complexing agent. Citric acid is one of the most widely distributed small molecule acids in nature. Liang et al. and Zhang et al. [17,18] found that the addition of citric acid can effectively control the amount of free ferrous ions in the system and ensure the continuous generation of free ferrous ions. The Fe^2+^ solution was made by adding FeSO_4_·7H_2_O and C_6_H_10_O_8_C_6_, according to the preliminary test; when the molar ratio of the Fe^2+^ to citric acid was 1:7, Fe^2+^ is oxidized the slowest. The experiments included two phases, in the first phase, it was a 45-day stabilization period, the bacterial communities were fed by 1/4 Hoagland nutrient solution, and were allowed to adapt nitrogen and phosphorus concentration in the wetland environment. The second phase (from 27 December 2020 to 4 February 2021) was divided 10 trials and each trial was 3 days. In the process of the experiment, firstly, 2 L of synthetic wastewater was added into the reactor in advance, and then 2 L of different concentrations of Fe^2+^ solution were poured into each reactor, and the control group was only tap water. To avoid causing a disturbance in the reactor, synthetic wastewater and Fe^2+^ solutions were prepared in two water tanks (70 L). The concentration before the simulated wastewater and simulated iron-containing groundwater entered the reactor is shown in Table 1. Moreover, before each inflow event, CWs were flushed with synthetic wastewater three times. The composition of the synthetic wastewater used in the stabilization period was the same as for the second phase.

### 2.3. Sample Collection and Determination

The effluent was sampled at the upper outlet and was measured at 20 min and 1, 4, 8, 24, 48, and 72 h during the first four trials of the second phase. Water quality indicators were not measured in the last six trials. The experimental result is the average of the previous three trials. All of the samples were filtered with a 0.45 um membrane (Navigator, Shanghai, China). The NH_4_^+^-N, NO_3_^−^-N, TP, Fe^2+^, total iron, and Fe^3+^ concentrations were determined using an Automatic Chemical Analyzer (Mode Smart Chem 200, Rome, Italy). At the end of the last trials, gravel substrate samples from the core of reactor were collected using a pre-sterilized 50 mL centrifuge tube and were frozen at −20 °C.

### 2.4. Microbial Community Analysis

Gravel substrate samples were sent to Sangon Biotech Co., Ltd. (Shanghai, China). The DNA extraction was performed using an E.Z.N.A. Soil DNA Kit (Omega, Norcross, Georgia, USA), following the manufacturer’s instructions. The 16S rRNA V3-V4 amplicon was amplified using KAPA HiFi Hot Start Ready Mix (2×) (TaKaRa Bio Inc., Otsu, Japan). Two universal bacterial 16S rRNA were amplified using primers 338F (5-ACTCCTACGGGAGGCAGCAG-3′) and 806R (5-GGACTACHVGGGTWTCTAAT-3′). The reaction was set up as follows: microbial DNA (10 ng /μL) 2 μL, amplicon PCR forward primer (10 μM) 1 μL, amplicon PCR reverse primer (10 μM) 1 μL, and 2 × KAPA HiFi Hot Start Ready Mix 15 μL (total 30 μL). We used AMPure XP beads to purify the free primers and primer dimer species in the amplicon product. Samples were delivered to Sangon Biotech (Shanghai, China) for library construction using universal Illumina adaptor and index. Sequencing was performed using the Illumina MiSeq system (Illumina MiSeq, Santiago, CA, USA), according to the manufacturer’s instructions.

Species richness and diversity statistics, including Coverage, Chao1, Ace, Simpson, and Shannon ever, were also calculated using Mothur V1.43.0 (Department of Microbiology & Immunology at The University of Michigan, Annaburg, MI, USA). Finally, all effective bacterial sequences without primers were submitted for downstream analysis.

Biological classification is based on the characteristics of organisms in terms of their morphological structure and physiological functions. The basic unit of classification is the species. The higher the classification level, the less the organisms have in common; the lower the classification level, the more the organisms have in common. The classification system is a hierarchical system, usually consisting of seven main levels: kingdom, phylum, class, order, family, genus, and species. Species (species) are the basic units, closely related species are grouped into genus, closely related genera are grouped into families, families belong to orders, orders belong to classes, classes belong to doors, and doors belong to kingdoms. The higher the classification level, the less the organisms have in common; the lower the classification level, the more the organisms have in common.

The database used in analysis was the RDP (Ribosomal Database Project) database (http://rdp.cme.msu.edu/index.jsp, accessed on 10 December 2021).

### 2.5. Statistical Analysis

Statistical analyses were performed using the SPSS 19.0 (SPSS Inc., Chicago, IL, USA). Data were presented as means ± standard deviation (SD). Means between different treatments were compared by one-way analysis of variance (ANOVA) with Tukey HSD test at a significance level of 0.05. All figures were designed and plotted by Origin 8.5 (OriginLab Inc., Northampton, MA, USA).

## 3. Results

### 3.1. Nutrients Removal Efficiency

**Figure 2:**
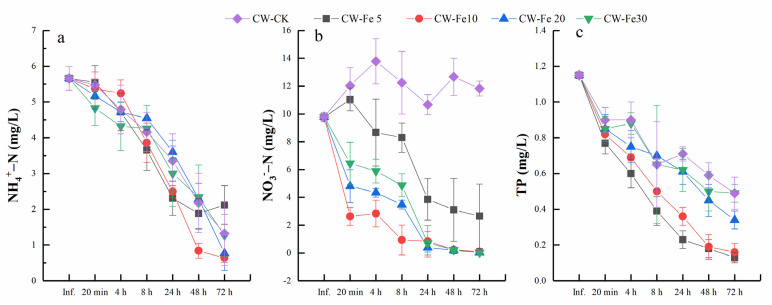
Changes of NH_4_^+^-N (**a**), NO_3_^−^-N (**b**), and TP (**c**) with HRT and influent Fe^2+^ concentrations.

The effluent concentrations of NH_4_^+^-N in CWs with different concentrations of Fe^2+^ additions are shown in Figure 2a. With the extension of HRT, the NH_4_^+^-N concentrations in the effluent water sharply decreased for a 72 h-HRT in all reactors. The removal efficiency of NH_4_^+^-N in the CW-Fe10 reactor was the highest, which reached 88.66%, it increased by 11.91% compared with the CW-CK, followed by CW-Fe20, CW-Fe30, and CW-Fe5, respectively. CW-Fe5 had the lowest removal rate (62.54%). The effluent concentrations of NH_4_^+^-N in both CW-Fe10 and CW-Fe20 at 72 h reached class III of the Surface Water Environmental Quality Standards of China (GB3838-2002). The removal efficiency of NH_4_^+^-N was 76.75% in CW-CK, it was consistent with the CW-Fe30 reactor at 72 h.

The effluent concentrations of NO_3_^−^-N in different reactors was shown in the Figure 2b. During the experiment period, compared with the CW-CK, the addition of Fe^2+^ had significantly (*p* < 0.05) less nitrate in the reactor. The highest NO_3_^−^-N removal rate was observed in CW-Fe10 at 8 h-HRT, which reached 90.36%, while the CW-Fe5, CW-Fe20, and CW-Fe30 were 15.08, 64.36, and 50.24%, respectively. NO_3_^−^-N removal rate in CW-Fe5, CW-Fe10, CW-Fe20, and CW-Fe30 increased by 45.49, 1.05, 31.69, and 42.60% at 24 h-HRT compared with 8 h-HRT, respectively. Except for CW-Fe5, the NO_3_^−^-N removal rates were all more than 91.40%, only it was 60.57–72.94% after 24 h. However, the removal rate of CW-Fe30 was slightly lower than the CW-Fe10 and CW-Fe20 after 24 h.

As shown in the Figure 2c, with the extension of HRT, the TP concentrations in effluent water sharply decreased in all of the reactors. It is worth noting that the effluent concentrations of CW-Fe5, CW-Fe10, and CW-Fe20 were significant (*p* < 0.05) lower than CW-CK after 4 h. There was no significant difference (*p* > 0.05) between CW-Fe30 and CW-CK, indicating that the addition of 30 m/L Fe^2+^ had little effect on the removal of TP. The highest TP removal rate was observed in CW-Fe5 (88.68%), followed by CW-Fe10 (85.92%), CW-Fe20 (70.46%), CW-Fe30 (57.69%), and CW-CK (57.06%) at 72 h, and the effluent concentration of TP in CW-Fe5 and CW-Fe10 reached class III of the Surface Water Environmental Quality Standards of China (GB3838-2002) at 72 h, and their concentrations were 0.13 mg/L and 0.16 mg/L, respectively. The TP removal efficiency of the reactor was expressed as CW-Fe5 > CW-Fe10 > CW-Fe20 > CW-Fe30 > CW-CK after 4 h. It shown that the removal rate of TP decreased with increasing the concentration of Fe^2+^ in the reactors. The optimal concentration of Fe^2+^ was 5 mg/L. The removal rate of TP in CW-Fe5 reached 80.08% with 24 h, so, in a short time, adding low concentrations of Fe^2+^ was beneficial to TP removal from the CWs.

### 3.2. Effluent Dissolved Fe^2+^, Total Iron and Fe^3+^, and pH Value Concentrations

**Figure 3:**
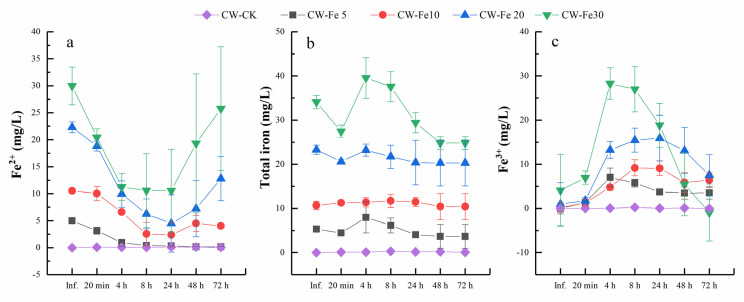
Changes of Fe^2^ (**a**), total iron (**b**), and Fe^3+^ (**c**) with HRT and influent Fe ^2+^ concentrations.

Furthermore, the concentrations of Fe^2+^, total iron, and Fe^3+^ were monitored (Figure 3), and the Fe^2+^ concentrations in effluent water sharply decreased between 20 min and 24 h, while they increased during 24 to 72 h, except for CW-Fe5. The concentrations of Fe^2+^ in CW-Fe5 decreased during the whole experiment. The effluent concentrations of Fe^2+^ in CW-Fe5, CW-Fe10, CW-Fe20, and CW-Fe30 were 0.32, 2.37, 4.48, and 10.57 mg/L at 24 h, respectively, while they were 0.15, 4.03, 12.78, and 25.76 mg/L at 72 h, respectively. The total iron in CW-Fe5, CW-Fe10, and CW-Fe20 maintained a stable and unchanging trend, and it shown a fluctuating trend in CW-Fe30. The concentrations of total iron in CW-Fe5, CW-Fe10, CW-Fe20, and CW-Fe30 were 4.04, 11.47, 20.38, and 29.41 mg/L at 24 h, respectively, while they were 3.66 mg/L, 10.42 mg/L, 20.30 mg/L, and 24.81 mg/L, respectively, 72 h. The Fe^3+^ concentrations in the effluent water increased slowly in CW-Fe10 and CW-Fe20 between 20 min and 24 h, while they decreased during 24 to 72 h, and increased in CW-Fe5 and CW-Fe30 between 20 min and 4 h, while decreasing during 4 to 72 h.

**Figure 4:**
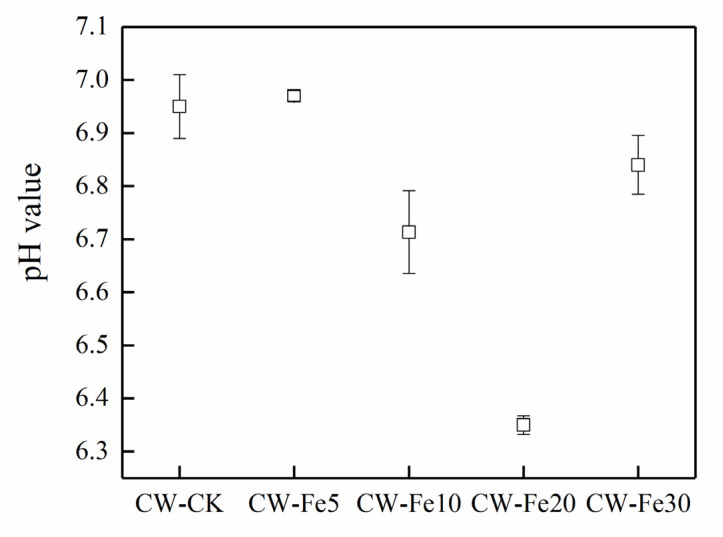
The pH value at 72 h.

The pH value (Figure 4) in CW-CK, CW-Fe5, CW-Fe10, CW-Fe20, and CW-Fe30 were 6.95, 6.97, 6.71, 6.35, and 6.84 at 72 h, respectively.

### 3.3. Microbial Community Characteristics in Substrates

#### 3.3.1. Microbial Richness and Diversity

Alpha diversity could reflect the abundance and diversity of microbial communities in locally homogeneous habitats, including a series of statistical analysis indexes, as shown in Table 2. OTUs were used to classify microbial species (e.g., species, genus, and phylum) artificially. The Chao1 richness index could estimate the number of microbial species. As a common indicator for assessing microbial diversity, the Shannon diversity index often reflects the species diversity and evenness of the community at the same time. The more evenly distributed the individuals, the higher the Shannon value. The higher the coverage rate, the lower the probability of undetected sequences. This index actually reflects whether the sequencing results represent the real situation of the samples. The Alfa diversity in all the reactors was well captured, with an estimated coverage of 0.99. The microbial coverage rate in the reactor without Fe^2+^ addition was 1.00. The diversity indexes of OTUs of the Shannon index for CW-Fe5, CW-Fe10, CW-Fe20, and CW-Fe30 were significantly (*p* < 0.05) lower than that of CW-CK. Chao1 in CW-Fe10, CW-Fe20, and CW-Fe30 were significantly (*p* < 0.05) lower than that of CW-CK. The diversity indexes of OTUs and Shannon in CW-Fe5 and CW-Fe10 were significantly higher (*p* < 0.05) than those of CW-Fe20 and CW-Fe30. It suggested that the addition of Fe^2+^ reduced the microbial diversity in the CWs. There was no significant difference of OTUs and Shannon between CW-Fe5 and CW-Fe10, and there was a significant difference (*p* < 0.05) of Shannon between CW-Fe20 and CW-Fe30. It can be inferred that when Fe^2+^ was higher than 20 mg/L in CWs, it could significantly reduce the microbial diversity in the reactor, thus indicating that 20 mg/L is the critical concentration for improving the microorganisms in CWs.

#### 3.3.2. Bacterial Community Composition

**Figure 5:**
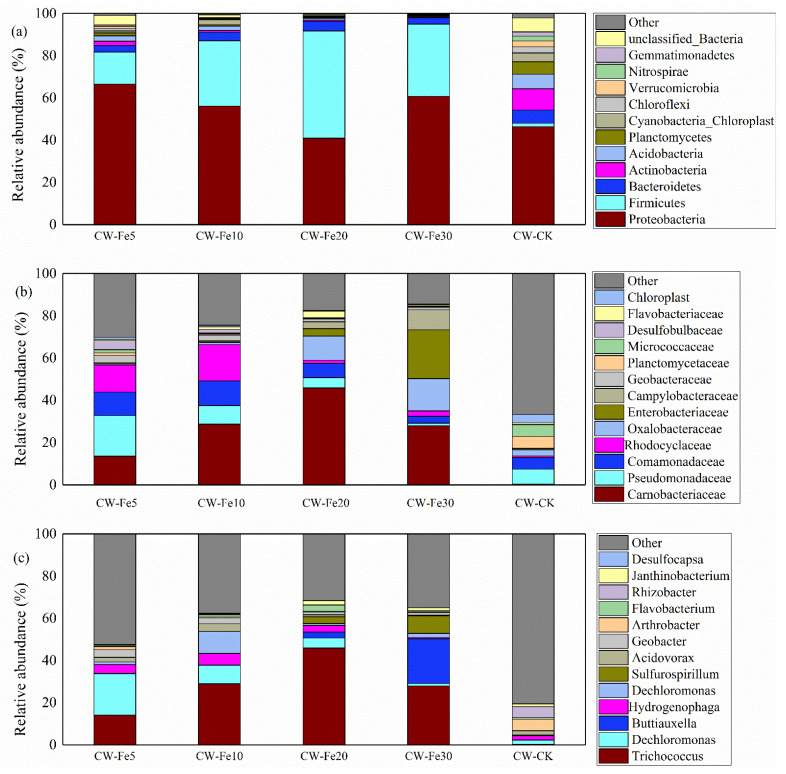
Relative abundance of microbial communities of different types of CW (phylum (**a**), family (**b**), and genus (**c**)).

The Figure 5 shown the microbial community composition and structure of each reactor substrates at the phylum (Figure 5a), family (Figure 5b), and genus (Figure 5c) levels. Phylum *Proteobacteria* accounted for the highest relative abundance at each sampling point, ranging from 40.89 to 66.57%. Compared with CW-CK, the addition of Fe^2+^ significantly (*p* < 0.05) increased the relative abundance of phylum *Firmicutes* in the reactors. The proportions of phylum *Bacteroidetes* found in CW-CK, CW-Fe5, CW-Fe10, CW-Fe20, and CW-Fe30 were 6.23, 3.08, 4.01, 4.64, and 3.04%, respectively. The relative abundances of phylum *Proteobacteria*, phylum *Firmicutes*, and phylum *Bacteroidetes* were the highest in CW-Fe5, CW-Fe20, and CW-CK, respectively.

At the family level, the major genera across the substrate samples of were detected as family *Carnobacteriaceae* (0.28–45.85%), family *Pseudomonadaceae* (1.20–19.15%), and family *Comamonadaceae* (3.32–11.67%). The proportion of family *Carnobacteriaceae* was the highest in CW-Fe20 (45.85%) and the lowest CW-CK (0.28%). Compared with the CK, the addition of Fe^2+^ significantly (*p* < 0.05) increased the relative abundance of family *Carnobacteriaceae* in the reactor. CW-Fe5 had the most family *Pseudomonadaceae* (19.15%), while it was difficult to identify in CW-Fe30 (1.20%). The relative abundance of family *Comamonadaceae* in CW-Fe5 (11.11%) and CW-Fe10 (11.66%) was significantly more than that in CW-CK (5.46%).

Meanwhile, at a genus level, the bacterial community structures of the substrates in five reactors were totally different. Genus *Trichococcus* in CW-Fe5, CW-Fe10, CW-Fe20, and CW-Fe30 were 13.55, 28.62, 45.85, and 27.89%, which is much more than that in CW-CK (0.28%). Compared with CW-CK, the addition of Fe^2+^ significantly (*p* < 0.05) increased the relative abundance of genus *Trichococcus* in the reactors. It was found that genus *Pseudomonas* dominated in the sample CW-CK (1.87%), CW-Fe5 (18.92%), CW-Fe10 (8.65%), CW-Fe20 (4.76%), and CW-Fe30 (1.15%). Genus *Buttiauxella* was abundant in CW-Fe30 (21.21%), while it contributed little to bacterial community in the other reactors (0.19–2.84%).

## 4. Discussion

### 4.1. Influence of Fe^2+^ Addition on Nitrogen Removal in CWs

NH_4_^+^-N concentrations in the effluent water sharply decreased for a 72 h-HRT in all reactors with the extension of HRT, and the removal rate of NH_4_^+^-N in CW-Fe10 was higher than for the other reactors. Some studies indicated that ammonia could be directly oxidized by O_2_ to NO_2_^−^, namely through nitrification [12]. Nitrification was the main reason for the reduction of NH_4_^+^-N concentrations in all reactors in this study, and it is a reason the NH_4_^+^-N concentrations in effluent water sharply decreased for a 72 h-HRT in all of the reactors. As an active element, iron participates in the nitrogen biogeochemical cycle [19]. Although the iron cycle only involves the conversion of two main chemical valences, they have significantly different influences on the biogeochemical cycle of nitrogen. A previous study demonstrated that the increase of ammonia monooxygenase (AMO) enzyme and nitrite oxidoreductase (NXR) in an Fe-added sludge system was conducive to nitrification [20]. In addition, the biochemical processes of oxidizing NH_4_^+^-N with Fe^3+^ (anaerobic ammonium oxidation coupled with ferric iron reduction (Feammox)) and reducing NO_3_^−^-N with Fe^2+^ (nitrate-dependent anaerobic ferrous-oxidation (NDAFO)) in an anaerobic environment are well known and have been found to coexist with Anammox. Feammox may occur in experimental reactors with Fe^2+^ addition. Studies have found that the process of Feammox has a better effect for sewage treatment in constructed wetlands, including a high removal rate in NH_4_^+^-N [12]; Scholars have also observed a positive correlation between dissolved iron, ammonium, and nitrite in three experimental wetlands [4]. Feammox needs a large amount of Fe (III) to improve nitrogen removal in wastewater treatment [12]. In this study, 53.62 to 78.81% Fe^2+^ was oxidized to Fe^3+^ in the CW-Fe20 and CW-Fe30 reactors after 24 h, resulting in higher Fe^3+^ concentrations (18.90 to 18.84 mg/L) and a large consumption of oxygen (anaerobic condition) in the reactors. In addition, phylum *Planctomycetes* contain bacteria related to Anammox [21]. Phylum *Planctomycetes* not only contribute to anammox, but also play an important role in denitrification [22]. Filamentous bacteria were detected in all reactors with Fe^2+^ addition, indicating that anammox may occur in the reactor, increasing the NH_4_^+^-N removal rate. It had a relatively higher concentration of Fe^3+^ in the CW-Fe20 and CW-Fe30 reactors, and there was also relatively high Fe^2+^ in the reactors. A high concentration of Fe^2+^ was not conducive to the oxidation of iron and ammonia, and would change the activity of the microorganisms. Studies have shown that facultative Fe^2+^ reducing bacteria may exist under both aerobic and hypoxic conditions in the transformation process of two iron forms. Fe^2+^ oxidation would affect the subsequent Fe^3+^ biological reduction by changing the bacterial activity. It is documented that microorganisms such as Gram-positive *Bacillus subtilis* variants, due to the production of reactive oxidants, under neutral conditions, air oxidation of Fe^2+^, and zerovalent iron could inactivate *Aspergillus niger* and Gram-negative *Escherichia coli* [23]. Therefore, Fe^2+^ oxidation may reduce the coexisting Fe^3+^ reducing bacteria, thus hindering the subsequent Fe^3+^ biological reduction [23]. After 24 h, Fe^3+^ was reduced to Fe^2+^, and Fe^2+^ increased in I20-CW and I30-CW in the reactors, which is not conducive to the removal of NH_4_^+^-N, nitrification, and Feammox. In addition, the chemical oxidation process of Fe^2+^ also consumed a large amount of O_2_, resulting in competition with nitrification of NH_4_^+^-N. Thus, it was difficult for microorganisms to oxidize NH_4_^+^-N, compared with chemical oxidation, which inhibited the NH_4_^+^-N removal [24].

In this study, the effluent removal efficiency of NH_4_^+^-N was 76.75% in CW-CK, and it was consistent with the CW-Fe30 reactor at 72 h. Some studies indicated that many processes, including nitrification, anammox, dissimilated nitrate reduction to ammonium (DNRA), substrate adsorption and desorption, denitrification, ammonia volatilization, and plant absorption, could affect NH_4_^+^-N removal in constructed wetlands [25]. In CW-CK, part of NH_4_^+^-N could be removed by nitrification, but Fe^2+^ was oxidized to Fe^3+^ in CW-Fe20 and CW-Fe30 and a large amount of oxygen was consumed, and nitrification was difficult to undertake. However, excessive Fe^2+^ concentrations led to a decrease in DO in the CWs system, thus inhibiting the activity of anammox bacteria and other microorganisms [26]. So, Fe^2+^ addition had less impact on the removal rate of NH_4_^+^-N, because more NH_4_^+^-N was generated during the denitrification process in the reactors. Some research has suggested that an increase in electron donors leads to a greater reduction of NO_3_^−^-N to N_2_ and NH_4_^+^-N instead of NO_2_^−^-N [15,27,28]. Si et al. [15] found that adding iron could cause the accumulation of NH_4_^+^-N, because it by produced Fe^2+^, thus providing more electron donors.

Compared with CW-CK, adding Fe^2+^ could significantly reduce the concentration of NO_3_^−^-N in CWs (*p* < 0.05). This indicates that Fe^2+^ promoted the denitrification process and participated in denitrification. This was consistent with previous relevant research conclusions [29]. Xu and Cai [30] added NO_3_^−^ and Fe^2+^ in subtropical soil for cultivation under anaerobic reduction conditions, and it was found that the NO_3_^−^ concentration was significantly positively correlated with the Fe^2+^ concentration, indicating that Fe^2+^ participated in the electron transfer process of denitrification. Zhang et al. [31] indicated that the removal rate of NO_3_^−^-N was 45.63 ± 3.31% in anaerobic digestion sludge with a paper inoculation source and Fe^2+^.

There are two mechanisms through which Fe^2+^ participated in the denitrification of CWs. On the one hand, Fe^2+^ donated electrons directly to nitrate [11]. However, under general liquid phase conditions, chemical denitrification did not occur easily or was very slow [32]. It promoted the solid interface or some solid materials, such as metal ions and metal oxide, to exist in chemical denitrification progress [33]. On the other hand, Fe^2+^ and NO_3_^−^-N depend on certain microorganisms for the reduction metabolism, which could reduce NO_3_^−^ while oxidizing Fe^2+^. In most cases, Fe^2+^ participating in the denitrification of NO_3_^−^-N was dominated by biological mechanisms. In addition, CWs was a comprehensive ecological treatment system with substrates and rich microorganisms, providing suitable conditions for chemical and biological denitrification [34]. With 20 min of influent, there was mainly chemical denitrification, and after 20 min, biological denitrification was dominant. If there was not sufficient organic carbon as an electron donor for heterotrophic denitrification in nitrate-dependent iron (II) oxidation, microorganisms could spontaneously utilize Fe^2+^ as an inorganic electron donor. Because of low organic carbon in reactors, the microorganisms would improve NO_3_^−^-N removal efficiencies through cooperation with Fe^2+^. The specific process is when Fe^2+^ is oxidized to Fe^3+^, NO_3_^−^-N is used as an electron donor, Fe^2+^ is oxidized to Fe^3+^, and NO_3_^−^-N is reduced to N_2_. In addition, the removal rate of NO_3_^−^-N was faster after 24 h in this study. The main reason was that the denitrification time increased with the prolongation of HRT, and there were more denitrifying bacteria after 24 h, and most nitrate-dependent iron (II) oxidizing strains relied on organic co-substrates, such as acetate [35]. In this study, some small molecular weight organic matter citric acid was added to maintain nitrate-dependent iron (II) oxidizing strains. Therefore, with the extension of HRT, the removal efficiency of NO_3_^−^-N increased. Third, the high concentration of Fe^2+^ continuously provided electron donors for biological denitrification. After 24 h of influent, the Fe^3+^ in the reactor was reduced to Fe^2+^. In these redox kinetic systems, Fe^2+^ could be oxidized by O_2_ to Fe^3+^ when O_2_ was depleted, the Fe^3+^ could be reduced to Fe^2+^ through the coexisting Fe^3+^ reducing bacteria. These redox transitions formed the Fe^2+^/Fe^3+^ cycles, continually providing electron donors for denitrification. This study also found that the removal rate of NO_3_^−^-N in CW-Fe20 and CW-Fe30 reactors was lower than that of CW-Fe10, which suggested that when the influent Fe^2+^ concentration was 10 mg/L, the removal rate of NO_3_^−^-N was the highest. The main reason may be the high concentrations of iron affected the activity of the microorganisms. Some studies have shown that iron could cause oxidative damage to microorganisms, thus leading to cell death [15].

### 4.2. Influence of Fe^2+^ Addition on Phosphorus Removal Performance in CWs

In this study, adding Fe^2+^ could increase the removal efficiency of TP. This was consistent with the conclusions of other research [25], and the concentration of Fe^2+^ was significantly negatively related with phosphorus concentrations in constructed wetland water. Iron enhanced the removal of phosphorus through abiotic and biological processes [36]. Similar research has shown that orthophosphate existed in domestic sewage and was removed by chemical precipitation and biological metabolism [20]. A low temperature has little effect on the removal of phosphorus, indicating that the non-biological process was the main way to remove phosphorus. From a non-biological point of view, the main reason was that iron promotes phosphorus removal through the following three mechanisms: coexistence and precipitation of Fe^2+^ or Fe^3+^, adsorption of solid iron oxide/hydroxide, and flocculation and precipitation of Fe^2+^ [25]. Firstly, when Fe^2+^ was added to the reactor, in a short time, the Fe^3+^ from the oxidation of Fe^2+^ reacted with the phosphate to form FeOOH [37] and Fe_3_(PO_4_)_2_ with a large specific surface area. Due to its low solubility in water, it was immediately precipitated as a solid [20]). Secondly, iron oxyhydroxide could be produced in reactors with Fe^2+^ addition, and the production of iron oxyhydroxide promoted the adsorption of phosphorus (iron oxyhydroxide−phosphate) [38]. The huge specific surface area of iron oxyhydroxide improved the adsorption capacity of iron filings. The adsorption of phosphorus by the substrate and plant roots are important ways to remove TP [14]. Both substrate and plant root absorption were found in the early stage of the experiments, and played an important role in TP removal [39]. Vymazal [40] found that most of the iron in the matrix was in the form of solid iron oxide, while ions were limited. Therefore, adsorption become the main method for phosphorus removal [41]. In this study, the phosphate was adsorbed by the matrix, which explains why the phosphate in CW-CK also had a certain removal rate. Finally, the phosphate could be removed by the formation of ferrous phosphate, deposited on the iron surface layer and not present in the precipitation agent [42]. The removal efficiency of TP decreased with the extension of HRT in this study. The results were consistent with the research of [14]. The main reason for this is that the gradual thickening of the biofilm on the substrate could hinder the effective contact and mass transfer between the pollutants and the substrate, resulting in a reduction of the removal efficiency of TP [43]. In addition, large Fe^2+^ could quickly consume oxygen in the wastewater, causing phosphorus-accumulating bacteria to release phosphorus under anaerobic conditions [44]. Meanwhile, due to low oxygen levels, Fe^3+^ was easily reduced to Fe^2+^, releasing complex phosphorus, thereby lowering the efficiency of phosphorus removal [45]. High concentrations of Fe^2+^ inhibited the removal efficiencies of TP, which may be attributed to the three main reasons. One was that excessive Fe^3+^ formed a large amount of iron oxide precipitation, covering the surface of the substrate, reducing the adsorption of phosphorus by the substrate. The other was that the addition of high concentration of Fe^2+^ could quickly result in an anaerobic environment in the reactors, and phosphorus accumulating bacteria release phosphorus [44]. The third is that, under anaerobic conditions, Fe^3+^ is easily reduced to Fe^2+^, which releases complex phosphorus, thereby reducing the efficiency of phosphorus removal [45]. Therefore, the higher concentration of Fe^2+^ inhibits the removal of TP.

### 4.3. Influence of Fe^2+^ Addition on the Features of Bacterial Communities

In this study, the iron addition reduced the microbial diversity in the reactors. This was consistent with the results of previous studies [46], which indicated that more Fe^2+^ led to a decrease in the abundance of microorganisms and an increase in the abundance of tolerant species. Moreover, the addition of Fe^2+^ could reduce the types of other bacteria and increase the relative abundance of specific dominant bacteria, thus enhancing nitrogen and phosphorus removal. Figure 5 shows the bacterial community structure of all five reactors at the phylum (Figure 5a), family (Figure 5b), and genus (Figure 5c) levels. Phylum *Proteobacteria* includes bacteria responsible for nitrification and denitrification activities and various metabolic bacteria. The circus diagram (Figure 5a) shows that phylum *Proteobacteria* accounted for the highest relative abundance at each sample, ranging from 40.89% to 66.57%. The existence of phylum *Proteobacteria* played an important role in the degradation of organic matter, nitrification and denitrification, and phosphorus removal in wastewater [47]. The *Alpha-proteobacteria*, *Beta-proteobacteria,* and *Gamma-proteobacteria* in the phylum *Proteobacteria* were also very important for sewage treatment. Phylum *Proteobacteria* was usually the largest phylum found in constructed wetland systems [48]. Iron-oxidizing microorganisms (Fe (II)-oxidizing microorganisms, FeOM) could oxidize Fe^2+^ to Fe^3+^ under aerobic or anoxic/anaerobic conditions, and thus synthesize organic matter [49]. From the perspective of the phylum, FeOM mainly included phylum *Nitrospira*, phylum *Firmicute,* phylum *Chlorobi,* and phylum *Proteobacteria* [49]. The related bacteria phyla were found, indicating that FeOM existed in the reactor. Previous research reported that nitrate-dependent iron oxidizing bacteria (NFeOB) were classified as autotrophic or heterotrophic microorganisms, and most of the isolated organisms belonged to phylum *Proteobacteria* and phylum *Actinobacteria* [50]. Phylum *Actinobacteria* played an indispensable role in the nitrogen cycle [47]. In this study, the abundance of phylum *Actinobacteria* and phylum *Proteobacteria* in CW-CK was higher, indicating that denitrification was the main pathway for the decrease of NO_3_^−^-N concentrations in CW-CK. Phylum *Firmicutes* were the dominant bacteria in most constructed wetlands. The proportion of phylum *Bacteroidetes* increased with the outbreak of cyanobacteria blooms, and gradually became the dominant bacteria in eutrophic waters [14]. Phylum *Bacteroidetes* were also related to denitrification, and were detected in all reactors. The abundance of phylum *Bacteroidetes* in CW-CK was higher than that in reactors with Fe^2+^ addition, and it could be deduced that t Fe^2+^ was not the key factor for the growth of phylum *Bacteroidetes*. This was consistent with the results of previous studies, where the relative abundance of phylum *Bacteroidetes* was inhibited by pyrite and ferrous sulfide substrates, whether in aeration or non-aeration areas [51]. The above studies show that adding Fe^2+^ resulted in less autotrophic denitrification, and heterotrophic denitrification or iron-dependent denitrification became dominant.

Figure 5b shows the bacterial community structure composition of the overall five CWs at the family level. The relative abundance of heterotrophic denitrifying bacteria (such as family *Comamonadaceae* and family *Planctomycetaceae*) were found in the reactors, and were higher in CW-Fe5 and CW-Fe10, closely related to the removal of nitrogen elements [52]. Some of the microorganisms were anammox bacteria, and it is suggested that, besides the conventional nitrogen element removal pathways, the anammox process may play an important role in the nitrogen transformation process of CWs [53]. Certain bacterial genera in the *Comamonadaceae* family (such as *Hydrogenophaga*) of the reactors have been proven to be a type of phosphorus accumulating bacteria, which could absorb the dissolved phosphorus in the water and convert it into polyphosphate in the body for energy accumulation. This is considered to be the main contributor to phosphorus removal [54]. A large number of studies have shown that some of the microorganisms in the *Comamonas* family (family *Comamonadaceae*) belong to denitrifying bacteria, and the relative abundance of such microorganisms is closely related to NO_3_^−^-N removal [55]. The higher NO_3_^−^-N removal efficiency in the CW-Fe5 and CW-Fe10 may be related to the relative abundance of the family *Comamonadaceae* and family *Planctomycetaceae*. Furthermore, a large number of phosphorus-accumulating bacteria and denitrifying phosphorus-accumulating bacteria also exist in the family *Comamonadaceae* [54]. It is beneficial to the removal of TP in CW-Fe5 and CW-Fe10 reactors. Family *Planctomycetes* not only had the function of anammox, but also contributed to denitrification [22]. Family *Planctomycetes* were detected in four CWs with Fe^2+^ addition, indicating that anammox may occur in the reactors. Studies have shown that autotrophic denitrification is the main reaction to remove nitrogen from pyrite and ferrous sulfide substrates, which is not conducive to the growth of the family *Planctomycetaceae* [14]. This also revealed that the relative abundance of family *Planctomycetes* in CW-CK was higher than for the other reactors in this study.

The structure and composition of the bacterial community in CWs at the genus level is shown in Figure 5c. The genus *Trichococcus*, within the family *Carnobacteriaceae,* the order *Lactobacillales*, affiliated to phylum *Firmicutes*, accounted for 13.55–45.85% of bacterium in the reactors with Fe^2+^ addition, while it only accounted for 0.28% of that in CW-CK. More specifically, genus *Trichococcus* was more abundant in the reactors with Fe^2+^ addition, and it was particularly enriched in CW-Fe20, so it is believed that adding Fe^2+^ was beneficial to genus *Trichococcus* growth. Tang et al. [56] suggested that genus *Trichococcus* belong to Fe (III)-reducing bacteria, and Baek et al. [57] indicated that genus *Trichococcus* was a kind of electroactive iron-reducing bacterium. They could reduce the oxidized Fe^3+^ to Fe^2+^ and continue to providing electrons for nitrate, further suggesting that genus *Trichococcus* may participate in nitrogen biodegradation. In addition, genus *Trichococcus* is a common acid-producing bacteria, mainly utilizing glucose to produce lactic acid, formic acid, acetic acid, and CO_2_ [58]. The enrichment of the genus *Trichococcus* was conducive to organic matter degradation, contributing to the utilization of carbon sources by heterotrophic denitrifiers [59]. At the same time, previous studies have shown that the genus *Trichococcus* in the constructed wetland with iron addition could achieve more efficient denitrification. Genus *Pseudomonas* is a genus of denitrifying bacteria and a dominant group of nitrifying bacteria in constructed wetlands. It played an important role in heterotrophic denitrification [14]. The *Geobacter* and *Pseusomonas* genera, derived from iron reducing bacteria, were important for NH_4_^+^-N oxidation and contributed to nitrogen removal [60]. *Pseudomonas* was beneficial for removing COD_cr_ and TN, and was the dominant genus for removing TP [9], which might be attributed to *Pseudomonas* as the weak Fe (III) reducers [60]. The *Pseudomonas* of CW-Fe5, CW-Fe10, CW-Fe20, and CW-Fe30 were 18.92%, 8.65%, 4.76%, and 1.15%, respectively, suggesting that it was an important factor in the removal of nitrogen and phosphorus.

## 5. Conclusions

By adding different concentrations of Fe^2+^ to the CWs, whether Fe^2+^ addition has a positive effect on nutrient removal was explored. The main conclusions are as follows: adding 10 mg/L Fe^2+^ has the best nitrogen removal rate, and adding 5 mg/L Fe^2+^ has the highest TP removal rate. The above low-concentrations Fe^2+^ are within the range of the Fe^2+^ concentrations in the groundwater in the Sanjiang Plain. Considering the field situation, a portion of groundwater can be pumped into wetlands in the Sanjiang Plain to reduce the nutrient concentrations in CWs. Compared with CW-CK, adding different concentrations of Fe^2+^ significantly (*p* < 0.05) reduces the microbial diversity in the reactor and increases the relative abundance of the genus *Trichococcus*, within the family *Carnobacteriaceae,* the order *Lactobacillales*, affiliated to phylum *Firmicutes*, which belongs to Fe (III)-reducing bacteria, and can reduce the oxidized Fe^3+^ to Fe^2+^ and continue to provide electrons for nitrate and achieve more efficient denitrification with a small number of denitrifying bacteria after Fe^2+^ addition.

## Figures and Tables

**Table 1:** The composition and concentration of the synthetic wastewater (mg/L).

	CW-CK	CW-Fe5	CW-Fe10	CW-Fe20	CW-Fe30
NH_4_Cl	10	10	10	10	10
KNO_3_	14	14	14	14	14
K_2_HPO_4_	2	2	2	2	2
FeSO_4_•7H_2_O	0	10	20	40	60
C_6_H_10_O_8_C_6_	0	262.68	525.35	1050.7	1576.05

**Table 2:** Summary statistics of microbial community diversity indices in CWs.

Systems	OTUs	Shannon	Chao1	Coverage
CW-CK	1454.67 ± 24.44 ^a^	5.74 ± 0.24 ^a^	1589.20 ± 65.24 ^a^	1.000 ± 0.001 ^a^
CW-Fe5	1255.34 ± 61.65 ^b^	4.26 ± 0.40 ^b^	1513.32 ± 82.05 ^a^	0.994 ± 0.001 ^b^
CW-Fe10	1114.33 ± 105.83 ^b^	3.66 ± 0.33 ^b^	1405.91 ± 130.45 ^ab^	0.994 ± 0.001 ^b^
CW-Fe20	914.00 ± 117.85 ^c^	2.92 ± 0.22 ^c^	1264.73 ± 127.85 ^bc^	0.993 ± 0.001 ^b^
CW-Fe30	797.33 ± 53.89 ^c^	2.72 ± 0.16 ^d^	1105.40 ± 122.96 ^c^	0.994 ± 0.001 ^b^

Note: The letters ^a–d^ indicate the significant difference in the chemical properties of gravel under different CWs.

## Data Availability

Not applicable.
